# Exploring the Role of Circadian Rhythm-Related Genes in the Identification of Sepsis Subtypes and the Construction of Diagnostic Models Based on RNA-seq and scRNA-seq

**DOI:** 10.3390/ijms26093993

**Published:** 2025-04-23

**Authors:** Xuesong Wang, Zhe Guo, Ziwen Wang, Xinrui Wang, Yuxiang Xia, Dishan Wu, Zhong Wang

**Affiliations:** Beijing Tsinghua Changgung Hospital, School of Clinical Medicine, Tsinghua University, Beijing 100084, China; wxs1103@mail.tsinghua.edu.cn (X.W.);

**Keywords:** sepsis, circadian rhythm-related genes, subtype identification, machine learning, scRNA-seq

## Abstract

Sepsis is a severe systemic response to infection that may lead to the dysfunction of multiple organ systems and may even be life-threatening. Circadian rhythm-related genes (CRDRGs) regulate the circadian clock and affect many physiological processes, including immune responses. In patients with sepsis, circadian rhythms may be disrupted, thus leading to problems such as immune responses. RNA-seq datasets of sepsis and control groups were downloaded from the Gene Expression Omnibus (GEO) database, and two sepsis subtypes were identified based on differentially expressed CRDRGs. Two gene modules related to sepsis diagnosis and subtypes were obtained using the weighted co-expression network (WGCNA) algorithm. Subsequently, using four machine learning algorithms (random forest, support vector machine, a generalized linear model, and xgboost), genes related to sepsis diagnosis were identified from the intersection genes of the two modules, and a diagnostic model was constructed. Single-cell sequencing (scRNA-seq) data were obtained from the GEO database to explore the expression landscape of diagnostic-related genes in different cell types. Finally, an RT-qPCR analysis of diagnosis-related genes confirmed the differences in expression trends between the two groups. Multiple differentially expressed CRDRGs were observed in the sepsis and control groups, and two subtypes were identified based on their expression levels. There were apparent differences in the distribution of samples of the two subtypes in two-dimensional space and the pathways involved. Using multiple machine learning algorithms, the intersection genes in the two most relevant modules of the WGCNA were identified, and a robust diagnostic model was constructed with five genes (*ARHGEF18, CHD3, PHC1, SFI1,* and *SPOCK2*). The AUC of this model reached 0.987 on the validation set, showing an excellent prediction performance. In this study, two sepsis subtypes were identified, and a sepsis diagnostic model was constructed via consensus clustering and machine learning algorithms. Five genes were identified as diagnostic markers for sepsis and can thus assist in clinical diagnosis and guide personalized treatment.

## 1. Introduction

Sepsis is a syndrome caused by the host’s abnormal response to infection. Most patients suffer from multiple organ dysfunction and failure, which may even be life-threatening [1,2]. With the continuous improvement of diagnosis and treatment, the mortality rate of sepsis has decreased significantly. However, long-term ICU hospitalization and sequelae place a heavy burden on patients. Early diagnosis and treatment can effectively delay the development of sepsis. Therefore, there is a need to identify further biomarkers and the subtypes of sepsis in order to improve the precise diagnosis and treatment of the condition [3,4,5].

The circadian rhythm refers to the periodic changes in an organism’s behavioral patterns and morphological structures over time. Previous studies have shown that an imbalance in the circadian rhythm is closely related to an increased risk of various diseases (cancer [6], diabetes [7], cardiovascular disease [8], etc.). Marpegan L. et al. found that, during the development of sepsis, mice injected intraperitoneally with a high dose of bacterial endotoxin lipopolysaccharide at the end of the resting phase of their activity had a higher mortality than those injected in the middle of the active stage [9]. Coiffard et al. found that the circadian rhythm of cytokines and leukocytes was impaired in patients with severe trauma [10]. In addition, because circadian rhythms can influence immune cell activity (leukocyte number and function), they may affect the intensity and timing of inflammatory responses in sepsis patients [11]. Therefore, further investigation into the correlation between circadian rhythms and sepsis is of significant importance, as it can provide more effective assistance in the early diagnosis of patients with sepsis, as well as in treatment and care strategies.

The aim of this study was to explore the critical role of circadian rhythm-related genes in the development of sepsis from a bioinformatics perspective. Specifically, the expression of circadian rhythm dysregulation-related genes (CRDRGs) was extracted from sepsis transcriptome data, and a differential expression analysis was performed. Consensus clustering based on differentially expressed genes (DEGs) was performed to identify sepsis subtypes. After extracting the most relevant gene modules in sepsis and control groups and different sepsis subtype groups based on a weighted co-expression network (WGCNA), diagnosis-related genes were identified, and a diagnostic model was built using various machine learning algorithms. Finally, the expression landscape of the diagnostic-related genes in different types of cell clusters was explored using single-cell sequencing (scRNA-seq) data.

## 2. Results

### 2.1. Obtaining and Analyzing the Results of Differentially Expressed Genes

**Figure 1:**
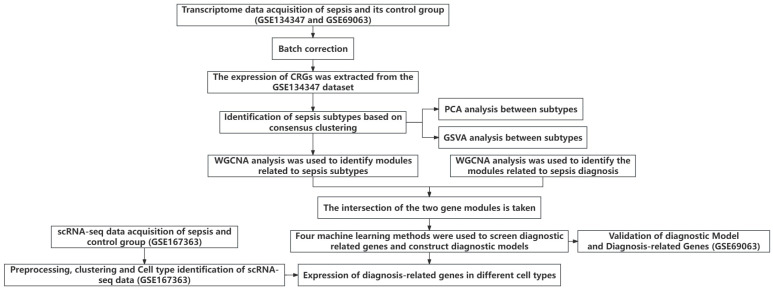
Overall flowchart.

**Figure 2:**
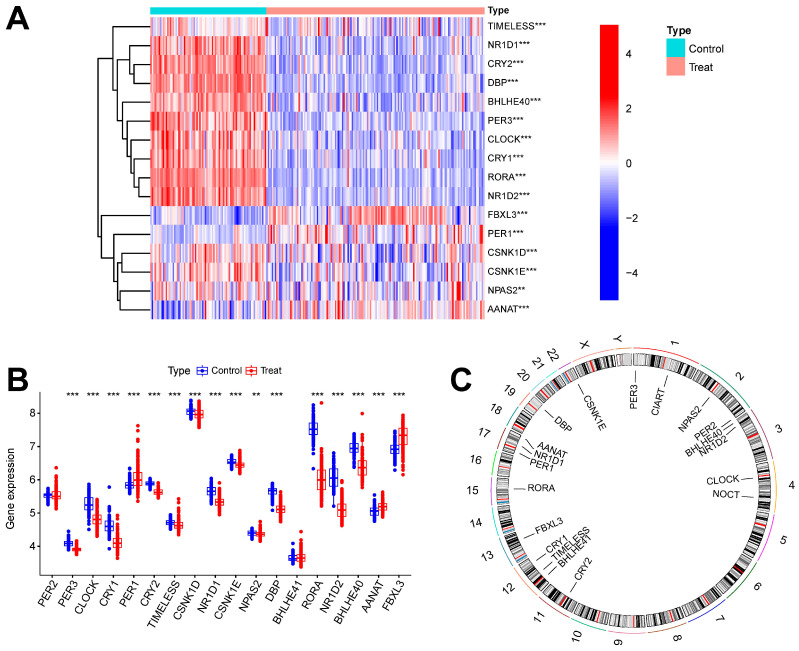

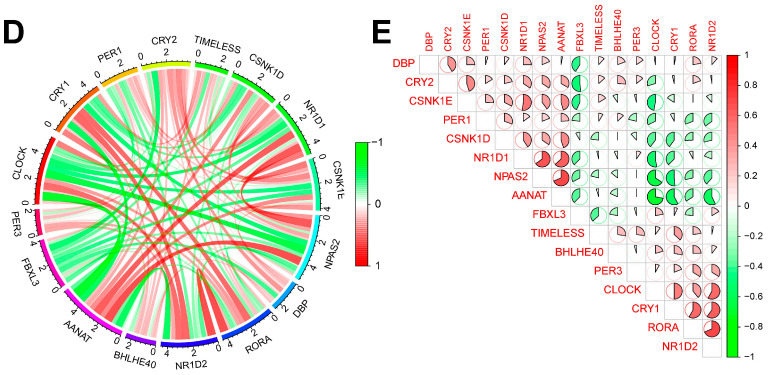
Batch correction and data identification of DEGs. (**A**) Expression heatmap of up- and downregulated DEGs. (**B**) Expression boxplot of up- and downregulated genes. (**C**) Circle diagram of DEGs at different positions on the chromosome. (**D**) Correlation circle diagram between DEGs. (**E**) Heatmap of correlations between DEGs. *** *p* less than 0.001; ** *p* less than 0.01.

Figure 1 shows the overall flowchart of this study. In this study, batch correction was first performed on the GSE134347 and GSE69063 datasets. CRGs were extracted from the GSE134347 dataset, and a differential expression analysis was performed. Sixteen DEGs demonstrated significant differences in expression (Figure 2A,B). After visualizing the locations of these genes on the chromosome (Figure 2C), a correlation analysis was performed on them. The Pearson correlations between most genes were significant (Figure 2D–E).

### 2.2. Identification of Sepsis Subtypes

**Figure 3:**
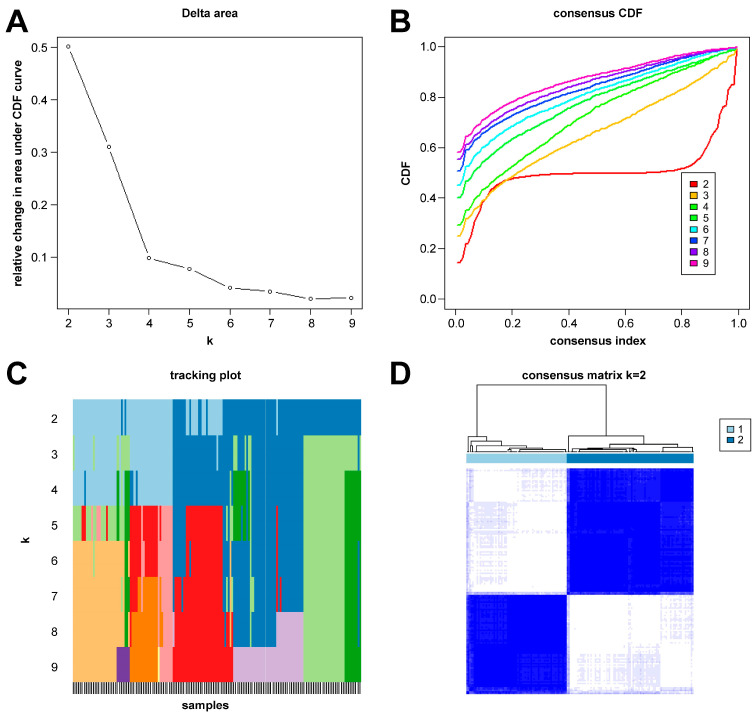
Results of sepsis subtype identification based on consensus clustering. (**A**) Consensus clustering CDF with k ranging from 2 to 9. (**B**) Cumulative distribution function (CDF) for consensus clustering results across different cluster numbers from 2 to 9, with each line representing a distinct cluster number, showing the proportion of samples that consistently cluster together across multiple runs of the algorithm. (**C**) Tracking plot constructed under different cluster numbers. The black stripes at the bottom of the picture represent the samples, showing the classification of the samples when k is 2–9. Blocks of different colors represent different categories. (**D**) Consensus matrix when k is 2.

**Figure 4:**
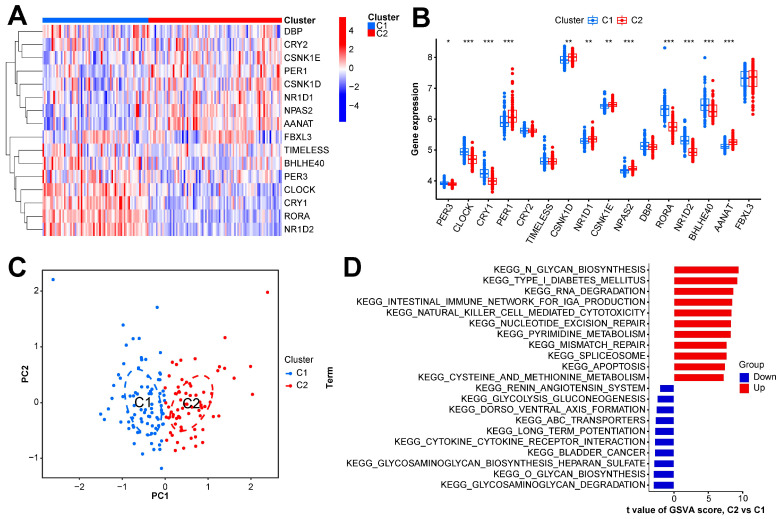
Expression and enrichment landscape of two sepsis subtypes. (**A**) Heatmap of expression of CRGs in the two subtypes. (**B**) Boxplot of the expression of CRGs in the two subtypes. (**C**) Scatter plot of samples obtained via PCA of the two subtypes. (**D**) GSVA result of the two subtypes. *** *p* < 0.001; ** *p* < 0.01; * *p* < 0.05.

In this study, we clustered the gene expression matrix of the 15 DEGs using the consensus clustering method. We set the number of clusters to 2–9. Figure 3A shows the relative changes in the area under the consistent cumulative distribution function (CDF) curve corresponding to different k values. Figure 3B shows the changes in the CDF at different k values. In addition, we also constructed a tracking plot corresponding to other cluster numbers (Figure 3C). The color changed less frequently when k was set to 2. After careful consideration, we finally set the number of clusters to 2 (confidence interval: 0.5625–0.8750). The expression of the CRGs significantly differed between the two subtypes (Figure 4A,B). We employed the “ConsensusClusterPlus” package in R software (4.2.3) to evaluate the stability of the clustering results under various parameter combinations (Table S2). The PCA found that the samples of the two subtypes could be clearly distinguished in the two-dimensional plane (Figure 4C). Differences between the two groups in terms of the upregulation and downregulation of DEGs were further analyzed using the “limma” algorithm (Supplementary Materials “diff_subtype.txt”). Under the conditions of p.adj < 0.05 and |logFC| > 0.5, a total of one significantly downregulated gene (RORA) was identified. Finally, we performed GSVA on the two groups of samples, aiming to identify differences in the biological pathways involved in the two subtypes (Figure 4D).

### 2.3. WGCNA Analysis Results

**Figure 5:**
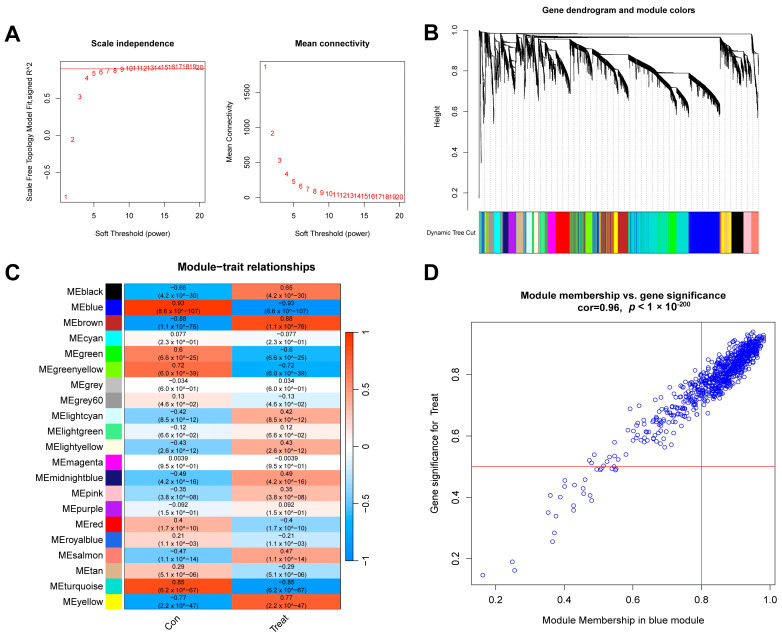
WGCNA analysis results of control and sepsis groups. (**A**) Scale-free fit index and average connectivity line chart obtained from WGCNA analysis. (**B**) Gene clustering tree. The branches at the top of the image represent genes, and the rectangles at the bottom of the image are made up of various modules. (**C**) Correlation heatmap of the relationship between modules and tags. (**D**) Scatter plot of correlation between blue modules and genes.

First, we conducted a WGCNA analysis based on the top 25% of the most fluctuating genes in the GSE134347 dataset. To ensure the overall connectivity of the co-expressed modules, the critical parameters were set as follows: “minModuleSize” was set to 50, and the soft threshold power was set to 6 (Figure 5A). Twenty-one co-expression modules were obtained. A color-coded gene clustering tree is shown in Figure 5B. Figure 5C shows that the blue module had the most significant correlation with the genes (*p* = 9 × 10^−107^, r = 0.93). Figure 5D shows a scatter plot of the correlation between the gene expression and sepsis samples in this module.

**Figure 6:**
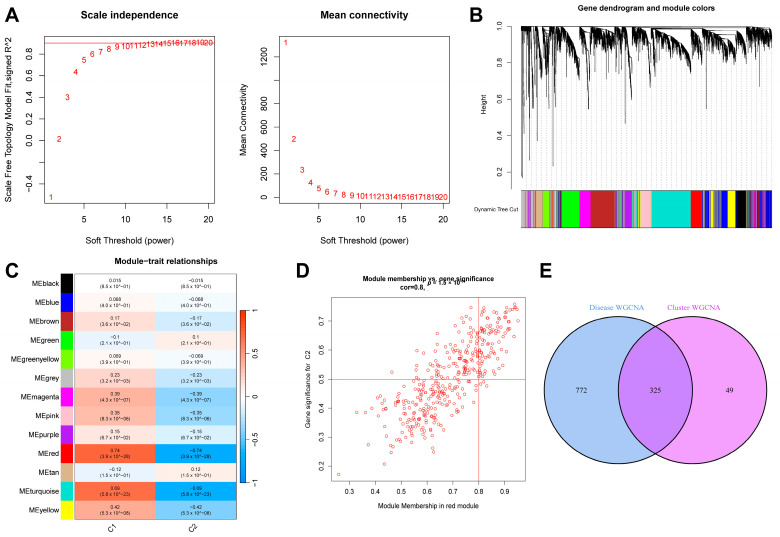
Results of WGCNA analysis of two sepsis subtypes. (**A**) Scale-free fit index and average connectivity line chart obtained from WGCNA analysis. (**B**) Gene clustering tree. (**C**) Correlation heatmap of the relationship between modules and tags. (**D**) Scatter plot of correlation between red modules and genes. (**E**) Intersection of genes from the two most correlated modules mentioned above.

Similarly, we extracted the sepsis samples from this dataset and selected the top 25% of genes with the most significant fluctuations for a WGCNA analysis. After setting “minModuleSize” to 100 and the critical parameter of the soft threshold power to 9 (Figure 6A), 13 co-expression modules were obtained. Figure 6B shows a gene clustering tree of the two subtypes. It can be seen in Figure 6C that the red module had the most significant correlation with the genes (*p* = 4 × 10^−26^, r = 0.74). Figure 6D shows a scatter plot of the correlation between the gene expression in this module and the C2 subtype samples. We obtained 325 genes after intersecting the genes in the above two most correlated modules (Figure 6E).

### 2.4. Construction of Sepsis Diagnostic Model and Verification Results

**Figure 7:**
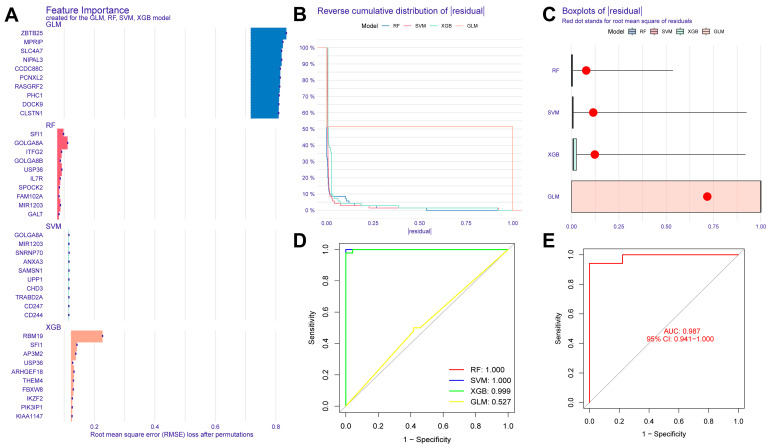
Evaluation results of diagnostic models built using four machine learning methods. (**A**) Contribution histogram of the top 10 important features obtained using the four methods. (**B**) Cumulative residual distribution plot of the four models. (**C**) Boxplot of the root mean square distribution of the residuals of the four models. (**D**) ROC analysis results obtained on the GSE134347 dataset utilizing the diagnostic model built using the four methods. (**E**) ROC analysis results obtained on the GSE69063 dataset utilizing the diagnostic model built using the xgboost method.

**Figure 8:**
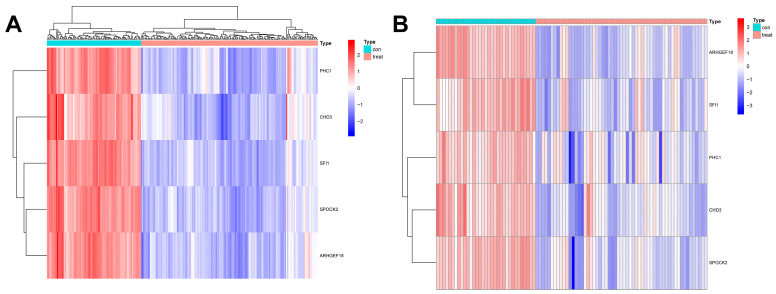

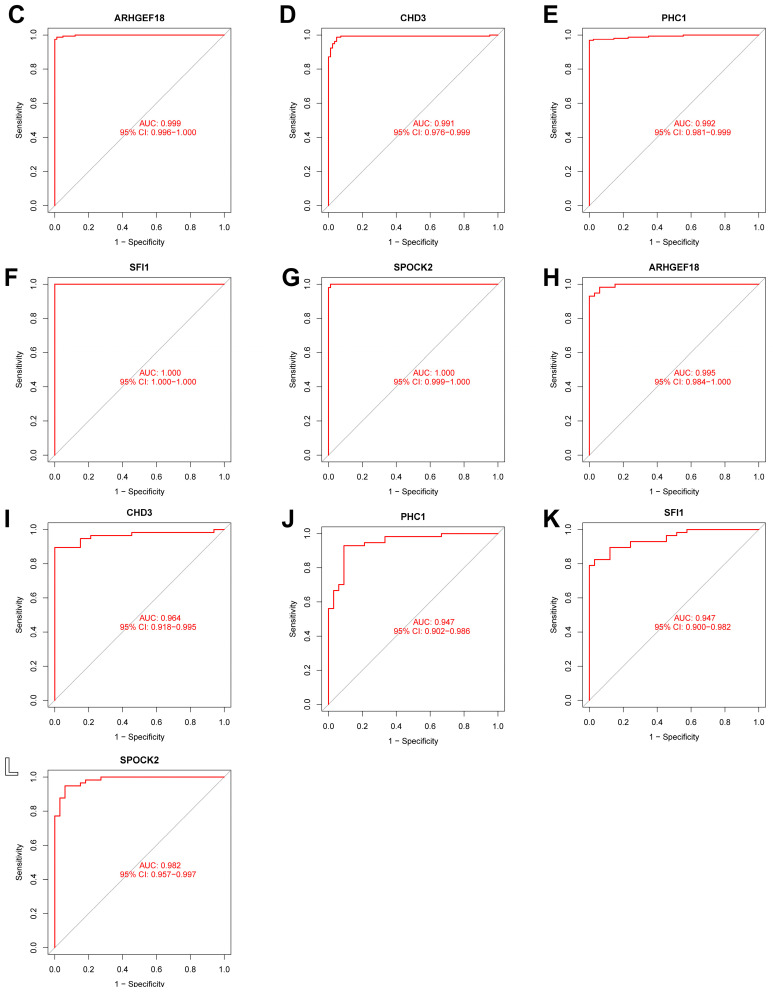
Analysis results obtained on the GSE69063 dataset utilizing the diagnostic model built using the xgboost method. (**A**,**B**) Expression heatmaps of diagnosis-related genes in the control and sepsis groups in the GSE134347 and GSE69063 datasets, respectively. (**C**–**G**) ROC curves of *ARHGEF18, CHD3, PHC1, SFI1,* and *SPOCK2* in the GSE134347 dataset. (**H**–**L**) ROC curves of *ARHGEF18, CHD3, PHC1, SFI1,* and *SPOCK2* in the GSE69063 dataset.

In this study, we used four machine learning methods (RF, SVM, xgboost, and GLM) to screen the 325 intersection genes obtained via the WGCNA analysis and build a sepsis diagnostic model. Figure 7A shows the top 10 most essential features selected by these algorithms in building the model. Among them, the xgboost algorithm had the lowest residual error, confirming that this algorithm has a better effect in building a diagnostic model. On the GSE134347 dataset, the ROCs of the four algorithms used to build diagnostic models were 1, 1, 1, and 0.492 (Figure 7B, Figure 7C, Figure 7D, and Figure 7E, respectively). The ROC of the xgboost algorithm with the lowest residual error reached 0.987 on the GSE69063 dataset, thus demonstrating a high prediction accuracy. To further confirm the effectiveness of the xgboost algorithm in constructing a diagnostic model, the five genes (*ARHGEF18, CHD3, PHC1, SFI1, and SPOCK2*) that constitute the model were separately analyzed. The expression of these genes significantly differed between the two groups (Figure 8A,B and Supplementary Materials Figure S2). The AUCs on the GSE134347 dataset were 0.999, 0.991, 0.992, 1, and 1, respectively (Figure 8C–G). The AUCs on the GSE69063 dataset were 0.995, 0.964, 0.947, 0.947, and 0.982, respectively (Figure 8H–L). In addition, the five genes had significant differences in expression between the two groups in both datasets (*p* < 0.05). In the external validation cohort (GSE13904 dataset), the AUC values of the five genes were 0.861, 0.759, 0.818, 0.812, and 0.837, respectively (Figure S3A and Figure S4). The diagnostic model constructed based on these five genes exhibited an AUC of 0.871, indicating a high level of diagnostic accuracy.

### 2.5. Construction Results of the Nomogram Model

**Figure 9:**
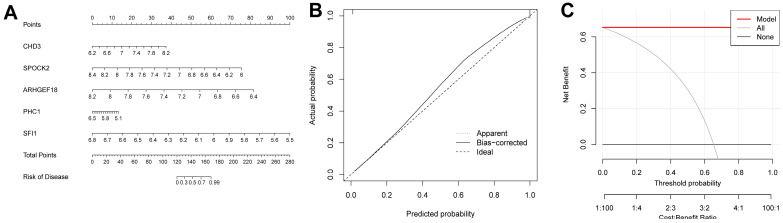
Construction results of the nomogram model. (**A**) Nomogram model constructed using diagnosis-related genes. (**B**) Calibration curve of the nomogram model. (**C**) DCA results.

We were able to intuitively understand the relationship between sepsis risk and the expression levels of the five FRGs by constructing a nomogram associated with the five genes (Figure 9A). A calibration curve was used to measure the predictive ability of the nomogram model (Figure 9B). In addition, we also introduced a DCA (Figure 9C). Its abscissa represents the threshold probability, and its ordinate represents the net benefit. Both the nomogram model and the nomogram were confirmed to have good reliability.

### 2.6. Analysis Results of scRNA-seq Data

**Figure 10:**
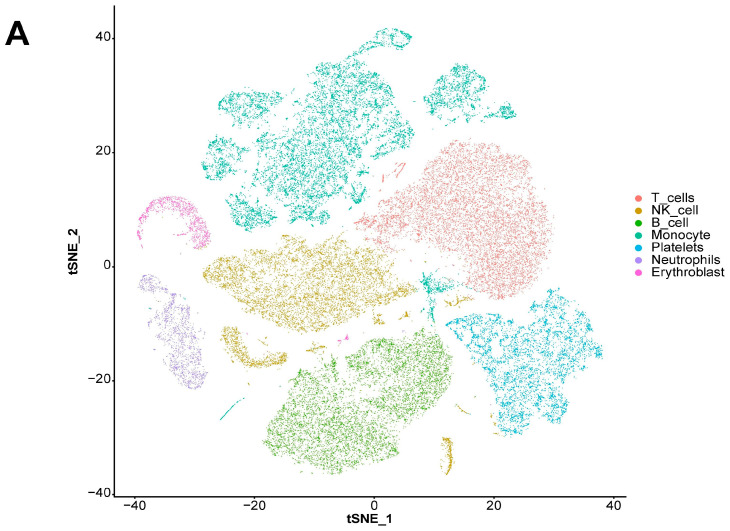

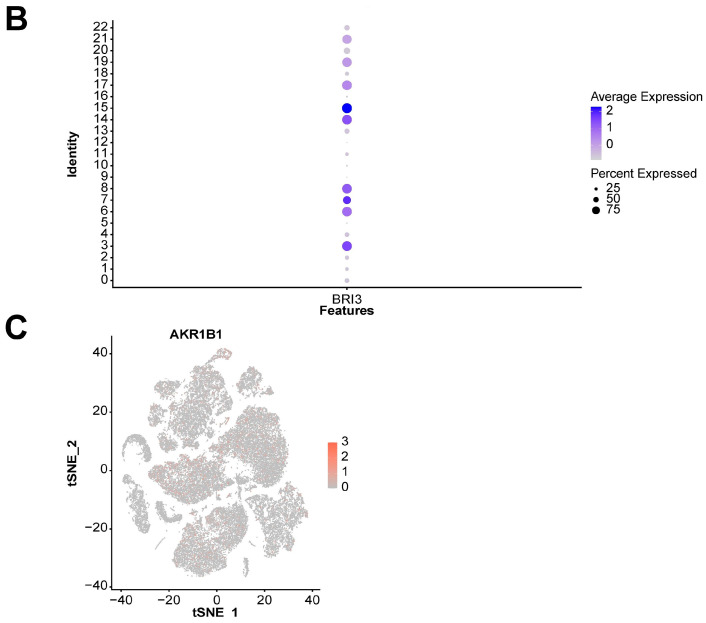
Analysis results of scRNA-seq data. (**A**) Visualization of TSNE dimensionality reduction for different cell types. (**B**) Bubble chart of the expression of five diagnostic-related genes in different cell types. (**C**) Heatmap of the expression of five diagnostic-related genes in different cell types.

We also explored the expression landscape of diagnostically relevant genes in different cell types (Figure 10). Specifically, we performed clustering and cell type annotation on the GSE167363 dataset (Figure S3) after preprocessing. Seven types of cells (T cells, NK cells, B cells, monocytes, platelets, neutrophils, and erythroblasts) were obtained. CHD3, SFI1, and SPOCK2 were found to be highly expressed in T cells.

### 2.7. Expression Validation of Diagnosis-Related Genes

**Figure 11:**
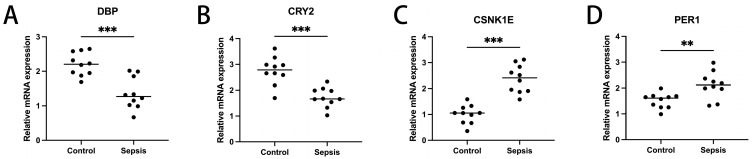

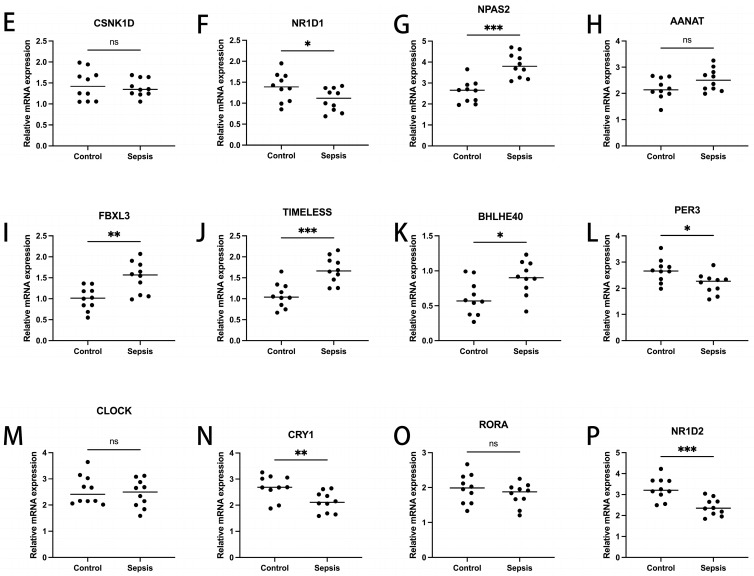
Transcriptional expression in whole blood from sepsis patients and healthy controls of DBP (**A**), CRY2 (**B**), CSNK1E (**C**), PER1 (**D**), CSNK1D (**E**), NR1D1 (**F**), NPAS2 (**G**), AANAT (**H**), FBXL3 (**I**), TIMELESS (**J**), BHLHE40 (**K**), PER3 (**L**), CLOCK (**M**), CRY1 (**N**), RORA (**O**), and NR1D2 (**P**), relative to GAPDH. Mean ± standard deviation. *** *p* less than 0.001; ** *p* less than 0.01; * *p* less than 0.05. ns: not significant.

To ascertain the expression profiles of sixteen diagnosis-related genes within the sepsis cohort versus control groups, we utilized quantitative reverse transcription polymerase chain reaction (qRT-PCR). Demographic data pertinent to this study are provided in Supplementary Table S1. Our findings revealed that, apart from CSNK1D, AANAT, CLOCK, and RORA, the expression levels of the other genes significantly differed between the two cohorts (Figure 11). These results align with the outcomes of our bioinformatics analysis. Therefore, further investigation into the roles of these genes in sepsis could lead to substantial advancements in the diagnosis and treatment of this condition.

## 3. Discussion

Sepsis is a systemic response to infection caused by bacteria, viruses, etc. As the disease progresses, it can lead to organ dysfunction, failure, etc. One of the main challenges currently faced regarding sepsis is the incompatibility between clinical practice and scientific research needs, leading to inconsistencies. Clinicians require the early and sensitive diagnosis of sepsis to develop appropriate clinical treatment strategies, and overdiagnosis is considered more important than precise diagnosis, as it avoids missing patients who may progress to sepsis [12]. Therefore, there is an increasing call for the early identification of suspected sepsis patients and early intervention [13]. In contrast, scientific research prioritizes the precision of collected data, with a reduced emphasis on patients at risk of developing sepsis, who are not the primary focus. This encompasses both patient and animal data, and such research can foster a deeper contemplation and discourse on the pathophysiological aspects of sepsis. As the central theme of this article indicates, there is a pressing need to identify more precise biomarkers associated with sepsis in order to enable earlier and more accurate diagnosis. However, the process of clinical translation is fraught with uncertainties. This is why, despite the introduction of the Sepsis 3.0 definition in 2016 [14], relevant research findings have not found widespread application in clinical practice. It is these types of challenges in sepsis research that continue to fuel scientists’ passion for exploration. Circadian rhythm-related genes are involved in the regulation of the biological clock, thereby triggering immune responses. Therefore, it is necessary to explore the role of CRDRGs in the development of sepsis.

First, in this study, we conducted a differential expression analysis based on the expression of CRDRGs in sepsis and control groups, and we obtained a total of 16 DEGs (PER1, PER3, CLOCK, CRY1, CRY2, TIMELESS, BMAL1, CSNK1D, NR1D1, CSNK1E, NPAS2, DBP, RORA, BHLHE40, AANAT, and FBXL3). Some DEGs have been confirmed to play a critical role in the development of sepsis. As clock genes, PER2, PER3, and CRY1 may be markers of sepsis severity [10]. In a study of circadian rhythms in septic shock, CRY2 and NR1D2 had the highest rhythmicity in patients [15]. Using mouse models, Sarah S. Geiger et al. found that BMAL1 signaling in hepatocytes is related to sepsis resistance [16]. Raffael et al. found that RORA coordinates with other genes in pediatric sepsis [17].

Second, we used the consensus clustering method to identify two sepsis subtypes based on the expression levels of DEGs. The two subtypes could be effectively distinguished in two-dimensional space and demonstrated significant differences in terms of participating pathways. Some pathways have been proven to be closely related to sepsis. NK cell-mediated immunity can be affected by reduced NK cell intrinsic receptor-mediated effects on viral ligands or infection [18]. Alternative splicing is a sepsis-related immune cell defect, and most genes related to AS are enriched in the apoptosis-related pathway.

Third, based on a WGCNA analysis, the most relevant significant gene modules were identified between the sepsis and control groups and the two sepsis subtypes. After taking the intersection of the two gene modules, the diagnosis-related genes (*ARHGEF18, CHD3, PHC1, SFI1, and SPOCK2*) were screened using four machine learning methods. Among them, the xgboost algorithm had the smallest residuals, and a diagnostic model was built using this method and externally verified. Interestingly, using machine learning methods, Fan et al. also identified *ARHGEF18* as a marker relevant to sepsis diagnosis [19]. The experimental results suggest that sepsis-related acute kidney injury may involve RAS disturbances, particularly the canonical angiotensin-converting enzyme angiotensin II/angiotensin II receptor 1 axis [20]. Studies have confirmed that hydrogen, mediated by PPARα and its regulation of ABC efflux transporters, reduces the permeability of the blood–brain barrier and can prevent brain dysfunction caused by sepsis [21].

The expression landscape of diagnostic-related genes in different types of cell clusters was analyzed using scRNA-seq datasets. *CHD3, SFI1*, and *SPOCK2* were found to be highly expressed in T cells. *SPOCK2* was also highly expressed in NK cells. Finally, our RT-qPCR results indicated that, with the exception of *CSNK1D, AANAT, CLOCK*, and *RORA*, the expression levels of the remaining genes significantly differed between the two cohorts (Figure 11). Regarding expression validation, the number of clinical samples that we collected was not sufficient, as this depends on the number of clinical patients and the duration of sample retention. At the same time, we plan to improve the comparison of gene expression levels in animal models related to sepsis, which will help to further support the reliability of the conclusions and enable an in-depth discussion from the perspective of diagnosis. Nevertheless, in light of the absence of a universally accepted and reliable model of sepsis in animal studies, the correlation between these animal models and patient outcomes has generated considerable debate and inspired innovative approaches [22]. We are closely monitoring the latest developments in this field of research. Consequently, we believe that it is not suitable to incorporate animal-related theoretical validations into this article at present. Nonetheless, we remain open to enhancing related experiments in the future.

## 4. Materials and Methods

### 4.1. Dataset Acquisition

RNA-seq and scRNA-seq data of sepsis and control groups were obtained from the Gene Expression Omnibus (GEO) database. Specifically, RNA-seq data from 83 control and 156 sepsis samples in the GSE134347 dataset were used to train the model. RNA-seq data from 33 control and 57 sepsis samples in the GSE69063 dataset were used to test the model. The GSE167363 dataset contains scRNA-seq data of human peripheral blood mononuclear cells from two control samples and ten sepsis samples, thus allowing for the exploration of the expression landscape of diagnostic-related genes in different cell types.

### 4.2. Batch Correction and Differential Expression Analysis

The R package “sva” was used to eliminate batch differences between the GSE134347 and GSE69063 datasets. After normalizing the expression data, 23 circadian-related genes (CRGs) were collected from the GeneCards database (https://www.genecards.org/, accessed on 10 july 2023). In the GSE134347 dataset, the R package (4.2.3) “limma” was employed, resulting in the identification of 15 DEGs that intersect with CRCs between the control and sepsis samples (Figure S1). A detailed list can be found in the “diff.csv” file of the Supplementary Materials. The threshold was set to *p* < 0.05. Additionally, we collected the transcriptome data of 209 sepsis cases and 18 controls from the GSE13904 dataset as an external validation cohort for the diagnostic genes and diagnostic models.

### 4.3. Subtype Identification and Analysis Methods

In this study, consensus clustering was used to identify sepsis subtypes. Specifically, the R package “ConsensusClusterPlus” was used to cluster sepsis based on DEGs. The number of clusters was set between 2 and 9, and the process was repeated 50 times. The clusterAlg parameter was set to “km” and the distance was set to “Euclidean”. Principal component analysis (PCA) was performed based on the expression levels of the DEGs, and principal components 1 and 2 were extracted as the X-axis and Y-axis, respectively. In addition, a gene set variation analysis (GSVA) of different subtypes was carried out based on the R package “GSVA”. The gmt file used in the study was c2.cp.kegg.symbols.gmt.

### 4.4. WGCNA Analysis

In this study, a WGCNA analysis was used to identify gene modules related to sepsis diagnosis/subtypes and to explore the relationship between gene networks and phenotypes. Specifically, the WGCNA algorithm first calculated the Pearson correlation coefficient between gene pairs and used its weighted value to establish connections between genes in a scale-free network. The soft threshold power is a crucial parameter in this process, and it determines the transformation of the correlation matrix into an adjacency matrix. In this study, the soft threshold power was selected based on the scale-free topology criterion and the algorithm’s recommendation. The “pickSoftThreshold” function was used to assess various power values (ranging from 1 to 20), and a power of 6 was chosen, as it provided a good balance between scale-free topology fit and mean connectivity, ensuring that the network was neither too dense nor too sparse. This selection was further validated by ensuring that the resulting network was biologically meaningful and stable. After determining the soft threshold power, a hierarchical clustering tree was constructed based on the correlation coefficients, and different gene modules were identified and color-coded. The parameter “minModuleSize” was set to 50 to ensure that only modules with a sufficient number of genes were considered. Module significance was calculated by correlating module eigengenes with the sepsis phenotype, and the correlation of mRNA expression levels with other modules was also assessed. Finally, the most significant modules related to the disease were identified, and characteristic genes were extracted for subsequent analysis. The above functions were implemented using the R package “WGCNA” (14). WGCNA is a powerful tool for identifying co-expressed gene modules, exploring the relationship between gene networks and phenotypes, and studying core genes in the network.

### 4.5. Construction of a Diagnostic Model Using Machine Learning Methods

Based on the R package “DALEX”, diagnostic models were constructed using random forest (RF), support vector machine (SVM), xgboost, and a generalized linear model (GLM). Specifically, the four algorithms were implemented by setting the method parameters in the train function provided by the package to “rf”, “svmRadial”, “xgbTree”, and “glm”, respectively. Furthermore, the explanation of the model was implemented using the explain and model_performance functions provided by the package. Finally, an ROC curve analysis of the diagnostic model was carried out using the R package “pROC”.

### 4.6. Nomogram Model and Decision Curve Analysis

The R packages “rms” and “rmda” were used to construct a nomogram model and perform a decision curve analysis (DCA) of diagnosis-related genes.

### 4.7. Analysis Methods of scRNA-seq Data

The preprocessing, clustering, and cell type identification of scRNA-seq data were mainly carried out using the R package “Seruat”. Specifically, after converting the original matrix into a Seurat object, cells with nFeature_RNA greater than 50 percent.mt less than five were retained. After log normalization, 1500 genes with more significant coefficients of variation between cells were extracted. After PCA, the first 15 principal components were included for a cluster analysis (the resolution parameter was set to 0.5). The cell types to which different clusters belonged were identified using the R package “singleR”.

*4.8. qRT-PCR*

Whole blood samples of ten patients with sepsis and ten healthy controls were collected from Beijing Tsinghua Changgung Hospital (Beijing, China). Their demographic information can be found in Table S1 in the Supplementary Materials. The screening criteria for the sepsis patients were based on Sepsis 3.0 (14). None of the patients had a history of autoimmune disorders, neoplastic diseases, or oral immunosuppressant use. Whole blood samples were obtained from the patients, and a quantitative real-time polymerase chain reaction (RT-PCR) was performed. This study was approved by the Ethics Committee (NCT05095324). The patients/participants provided their written informed consent to participate in this study. All experiments were performed in accordance with sepsis guidelines and regulations.

## 5. Conclusions

In summary, the identification of two sepsis subtypes through the consensus clustering of differentially expressed CRDRGs provides a basis for the stratified treatment of sepsis patients. The robust diagnostic model constructed using four machine learning algorithms and based on five CRDRGs (*ARHGEF18, CHD3, PHC1, SFI1,* and *SPOCK2*) demonstrates an excellent prediction performance, with an AUC of 0.987 on the validation set. These findings provide not only valuable insights into the underlying pathophysiology of sepsis but also potential diagnostic markers and therapeutic targets. Future work should focus on validating these markers in larger cohorts and exploring their roles in targeted therapies for sepsis.

## Data Availability

The original data presented in this study are included in the article/Supplementary Materials. Further inquiries can be directed to the corresponding author.

## Supplementary Materials

The following supporting information can be downloaded at https://www.mdpi.com/article/10.3390/ijms26093993/s1.

## References

[1] Casserly B., Phillips G.S., Schorr C., Dellinger R.P., Townsend S.R., Osborn T.M., Reinhart K., Selvakumar N., Levy M.M. (2015). Lactate measurements in sepsis-induced tissue hypoperfusion: Results from the Surviving Sepsis Campaign database. Crit. Care Med..

[2] Rudd K.E., Johnson S.C., Agesa K.M., Shackelford K.A., Tsoi D., Kievlan D.R., Colombara D.V., Ikuta K.S., Kissoon N., Finfer S. (2020). Global, regional, and national sepsis incidence and mortality, 1990–2017: Analysis for the Global Burden of Disease Study. Lancet.

[3] Shu Q., She H., Chen X., Zhong L., Zhu J., Fang L. (2023). Identification and experimental validation of mitochondria-related genes biomarkers associated with immune infiltration for sepsis. Front. Immunol..

[4] Jiang H., Ren Y., Yu J., Hu S., Zhang J. (2023). Analysis of lactate metabolism-related genes and their association with immune infiltration in septic shock via bioinformatics method. Front. Genet..

[5] Zheng Y., Wang J., Ling Z., Zhang J., Zeng Y., Wang K., Zhang Y., Nong L., Sang L., Xu Y. (2023). A diagnostic model for sepsis-induced acute lung injury using a consensus machine learning approach and its therapeutic implications. J. Transl. Med..

[6] Sancar A., Lindsey-Boltz L.A., Gaddameedhi S., Selby C.P., Ye R., Chiou Y.-Y., Kemp N.G., Hu J., Lee J.H., Ozturk N. (2015). Circadian clock, cancer, and chemotherapy. Biochemistry.

[7] Mason I.C., Qian J., Adler G.K., Scheer F.A.J.L. (2020). Impact of circadian disruption on glucose metabolism: Implications for type 2 diabetes. Diabetologia.

[8] Montaigne D., Marechal X., Modine T., Coisne A., Mouton S., Fayad G., Ninni S., Klein C., Ortmans S., Seunes C. (2018). Daytime variation of perioperative myocardial injury in cardiac surgery and its prevention by Rev-Erbα antagonism: A single-centre propensity-matched cohort study and a randomised study. Lancet.

[9] Marpegan L., Leone M.J., Katz M.E., Sobrero P.M., Bekinstein T.A., Golombek D.A. (2009). Diurnal variation in endotoxin-induced mortality in mice: Correlation with proinflammatory factors. Chronobiol. Int..

[10] Coiffard B., Diallo A.B., Culver A., Mezouar S., Hammad E., Vigne C., Nicolino-Brunet C., Dignat-George F., Baumstarck K., Boucekine M. (2019). Circadian Rhythm Disruption and Sepsis in Severe Trauma Patients. Shock.

[11] Scheiermann C., Gibbs J., Ince L., Loudon A. (2018). Clocking in to immunity. Nat. Rev. Immunol..

[12] De Backer D., Deutschman C.S., Hellman J., Myatra S.N., Ostermann M., Prescott H.C., Talmor D., Antonelli M., Pontes Azevedo L.C., Bauer S.R. (2024). Surviving Sepsis Campaign Research Priorities 2023. Crit. Care Med..

[13] Wang X., Guo Z., Chai Y., Wang Z., Liao H., Wang Z., Wang Z. (2023). Application Prospect of the SOFA Score and Related Modification Research Progress in Sepsis. J. Clin. Med..

[14] Singer M., Deutschman C.S., Seymour C.W., Shankar-Hari M., Annane D., Bauer M., Bellomo R., Bernard G.R., Chiche J.-D., Coopersmith C.M. (2016). The third international consensus definitions for sepsis and septic shock (Sepsis-3). JAMA.

[15] Lachmann G., Ananthasubramaniam B., Wünsch V.A., Scherfig L.M., Von Haefen C., Knaak C., Edel A., Ehlen L., Koller B., Goldmann A. (2021). Circadian rhythms in septic shock patients. Ann. Intensive Care.

[16] Geiger S.S., Traba J., Richoz N., Farley T.K., Brooks S.R., Petermann F., Wang L., Gonzalez F.J., Sack M.N., Siegel R.M. (2021). Feeding-induced resistance to acute lethal sepsis is dependent on hepatic BMAL1 and FXR signalling. Nat. Commun..

[17] Oliveira R.A.C., Imparato D.O., Fernandes V.G.S., Cavalcante J.V.F., Albanus R.D., Dalmolin R.J.S. (2021). Reverse Engineering of the Pediatric Sepsis Regulatory Network and Identification of Master Regulators. Biomedicines.

[18] Jensen I.J., Winborn C.S., Fosdick M.G., Shao P., Tremblay M.M., Shan Q., Tripathy S.K., Snyder C.M., Xue H.-H., Griffith T.S. (2018). Polymicrobial sepsis influences NK-cell-mediated immunity by diminishing NK-cell-intrinsic receptor-mediated effector responses to viral ligands or infections. PLoS Pathog..

[19] Fan Y., Han Q., Li J., Ye G., Zhang X., Xu T., Li H. (2022). Revealing potential diagnostic gene biomarkers of septic shock based on machine learning analysis. BMC Infect. Dis..

[20] Garcia B., Zarbock A., Bellomo R., Legrand M. (2023). The role of renin-angiotensin system in sepsis-associated acute kidney injury: Mechanisms and therapeutic implications. Curr. Opin. Crit. Care.

[21] Bai Y., Mi W., Meng X., Dong B., Jiang Y., Lu Y., Yu Y. (2023). Hydrogen alleviated cognitive impairment and blood-brain barrier damage in sepsis-associated encephalopathy by regulating ABC efflux transporters in a PPARα-dependent manner. BMC Neurosci..

[22] Cortellini S., DeClue A.E., Giunti M., Goggs R., Hopper K., Menard J.M., Rabelo R.C., Rozanski E.A., Sharp C.R., Silverstein D.C. (2024). Defining sepsis in small animals. J. Vet. Emerg. Crit. Care.

