# Supplementary material for: Exploring the Role of Circadian Rhythm-Related Genes in the Identification of Sepsis Subtypes and the Construction of Diagnostic Models Based on RNA-seq and scRNA-seq
Source: Int J Mol Sci. 2025 Apr 23;26(9):3993. doi: 10.3390/ijms26093993 (PMC12071451; doi:10.3390/ijms26093993)
Supplement: Supplementary file 1 [file ijms-26-03993-s001.zip › ijms-3556792-supplementary.pdf]

Supplementary Material

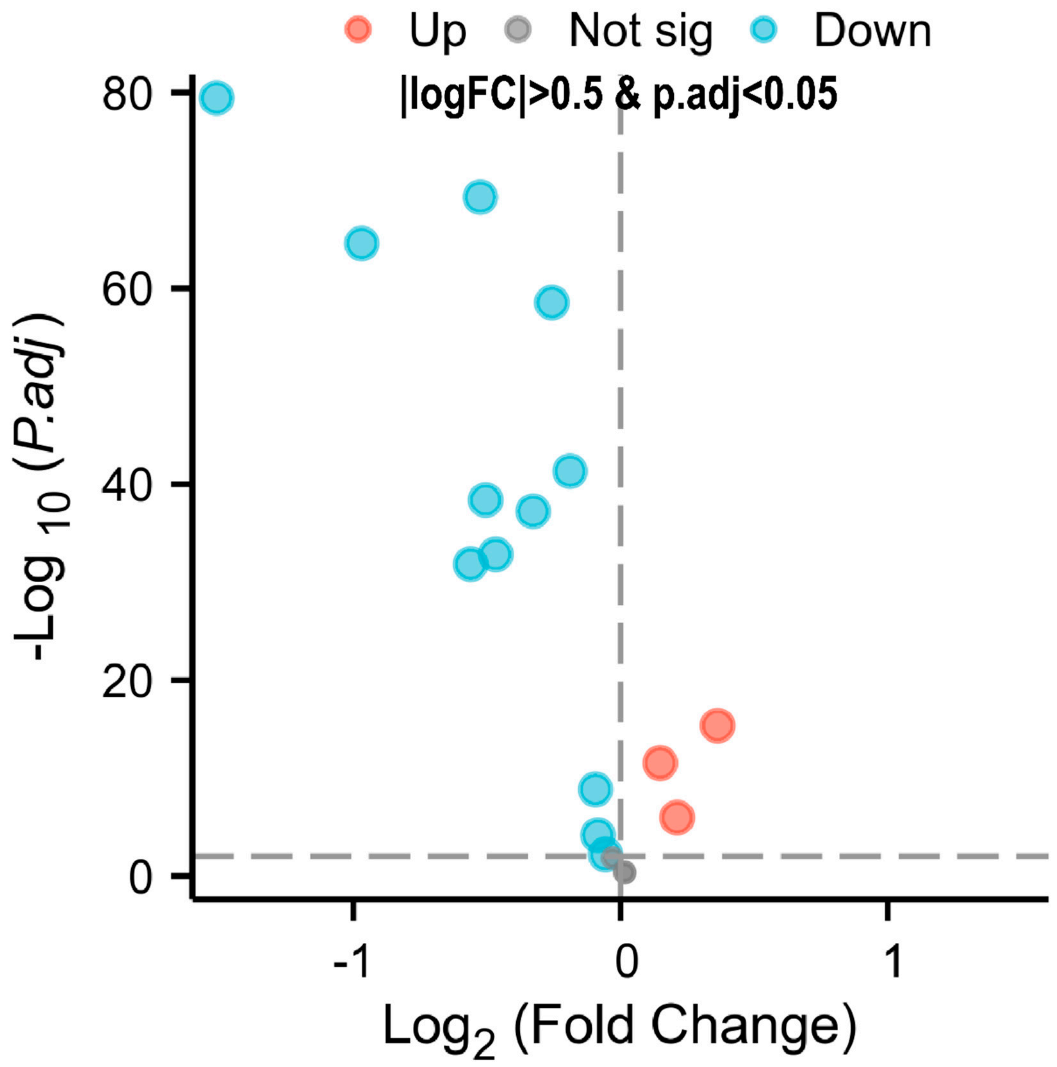

Figure S1. Volcano map obtained from intersection gene differential expression analysis.

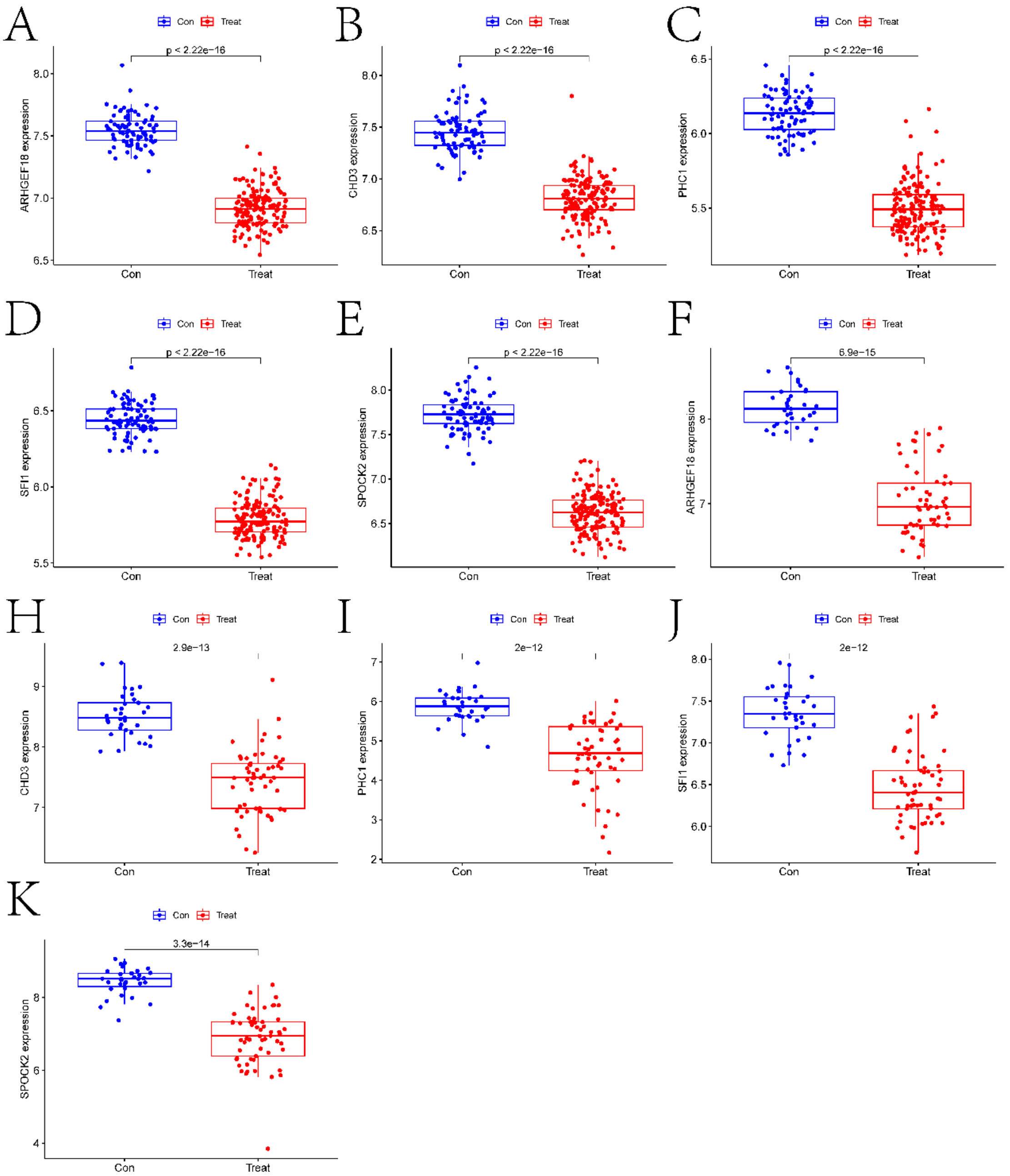

Figure S2. Expression boxplot of diagnostic-related genes. A-E are box plots of the expression of *ARHGEF18*, *CHD3*, *PHC1*, *SFI1* and *SPOCK2* in the control group and sepsis group in the GSE134347 data set respectively. F-K are box plots of the expression of *ARHGEF18*, *CHD3*, *PHC1*, *SFI1* and *SPOCK2* in the control group and sepsis group in the GSE69063 data set respectively.

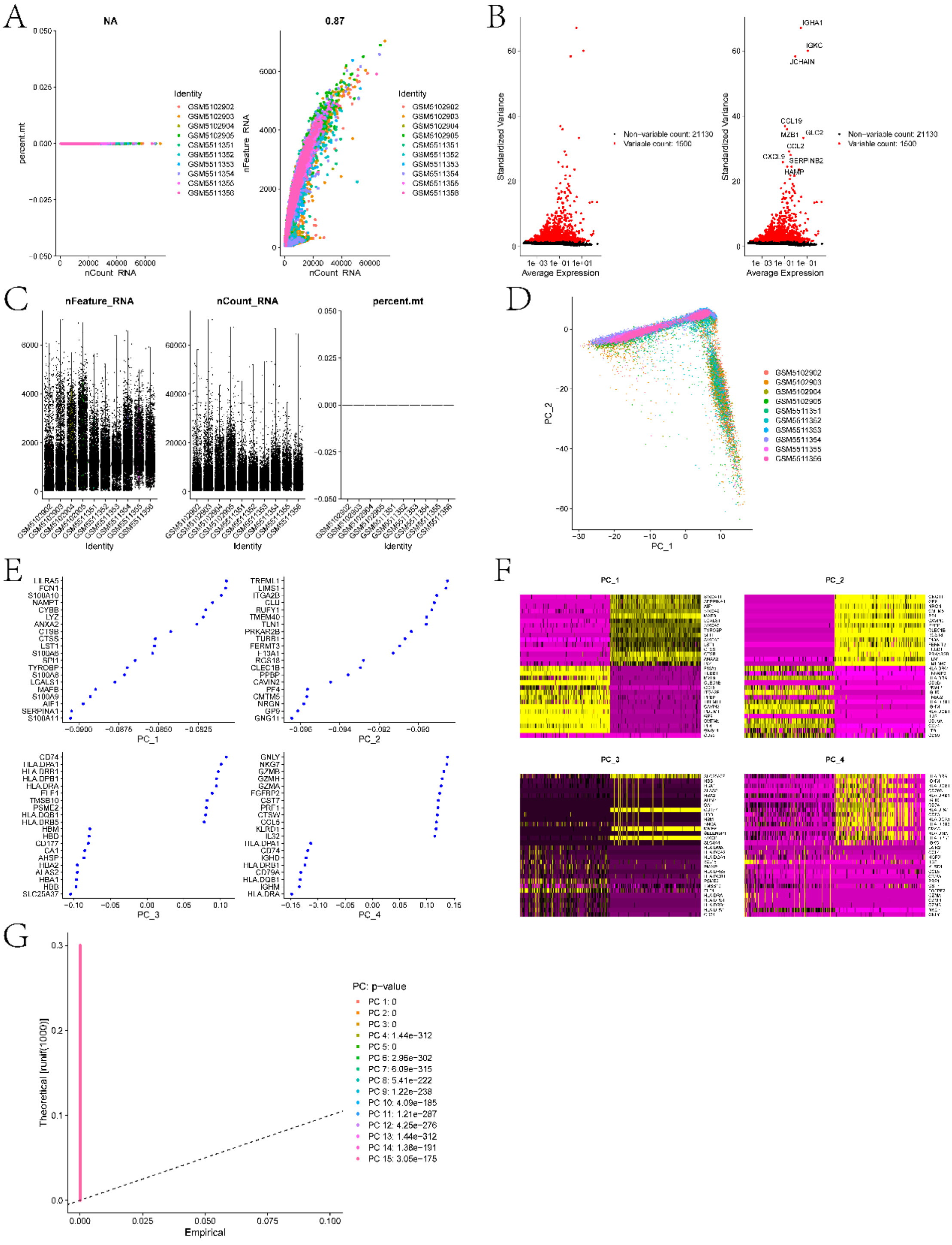

Figure S3. Preprocessing of scRNA-seq data. A is a scatter plot of the correlation between the sum of the expression levels of all genes detected in each cell and the proportion of mitochondrial genes/the number of genes detected in each cell. B is the screening result of hypervariable genes. C is a violin plot of the number of genes detected in each cell in the sample, the sum of the expression levels of all genes, and the proportion of mitochondrial genes. D is a scatter plot of different cells in two-dimensional space. E is a bubble plot of the top 20 genes in the first four principal components. F is the expression heat map of the top 20 genes in the first four principal components. G is the firework plot of PCA analysis.

Table S1. Demographic information for the Sepsis group and the Control group.

Parameters	Control	Sepsis	*P*
**Gender**			0.653
Male	6	5	
Female	4	5	
**Ages**			0.001
≥60	1	7	
18-60	9	3	
≤18	0	0	
**Infection Source**			N
Lung	0	6	
Urinary tract	0	1	
Skin or Soft tissues	0	0	
Other	0	3	
**Comorbidities**			0.001
Yes	0	9	
No	10	1	
**ΔSOFA Scores**			0.001
0-2	10	0	
≥2	0	10	
**Lactate levels[a] (mmol/L)**	0.61±0.23	4.09±1.89	0.001

[a] Lactate levels: Mean ± standard deviation, measurement time point: Sepsis group: within 24h after diagnosis of sepsis; Control group: the day of physical examination.

Table S2. The profile coefficients of *ConsensusClusterPlus* function with different parameters.

Parameter Condition	Profile Coefficient
ClusterAlg="km"; distance="euclidean"	0.588
ClusterAlg="hc"; distance="binary"	-0.137
ClusterAlg="hc"; distance="canberra"	0.197
ClusterAlg="hc"; distance="euclidean"	0.197
ClusterAlg="hc"; distance="maximum"	0.197
ClusterAlg="hc"; distance="minkowski"	0.197

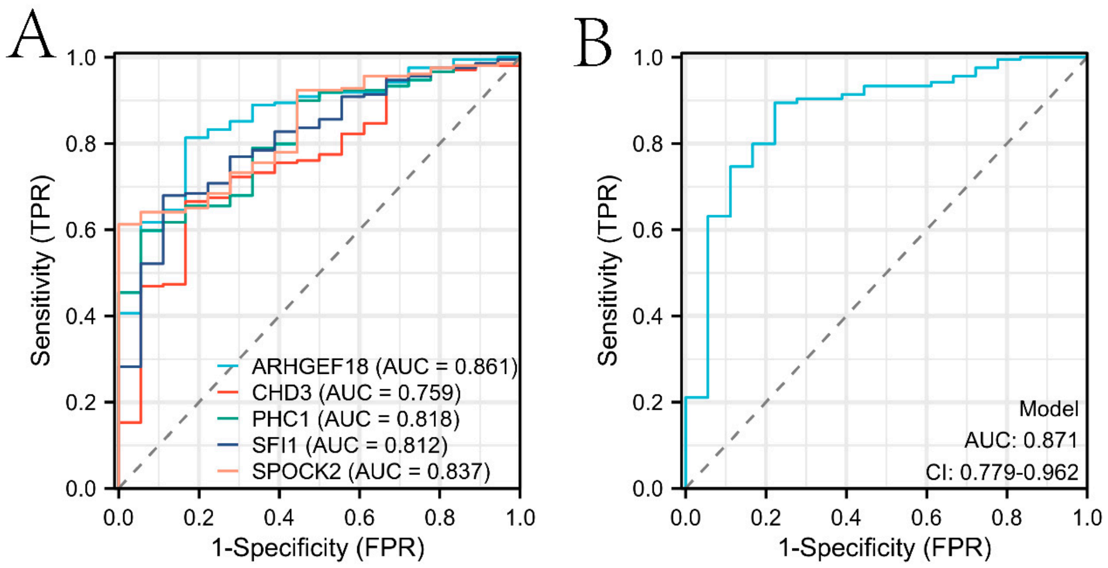

Figure S4. ROC Curves of Five Diagnostic Genes and the Diagnostic Model in the GSE13904 Dataset. (A) ROC curves of the five diagnostic genes. (B) ROC curve of the diagnostic model.
